# Supplementary figures and images for: Synergistic Antitumor Effect of Combined Radiotherapy and Engineered Salmonella typhimurium in an Intracranial Sarcoma Mouse Model
Source: Vaccines (Basel). 2023 Jul 23;11(7):1275. doi: 10.3390/vaccines11071275 (PMC10385126; doi:10.3390/vaccines11071275)

Day 4

Mouse 1

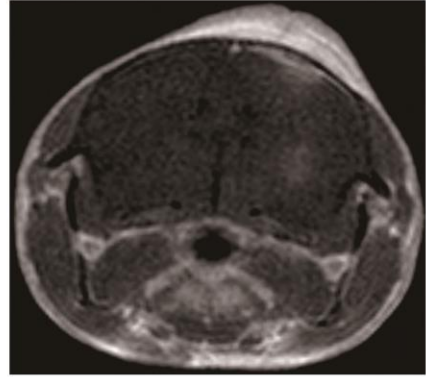

Mouse 2

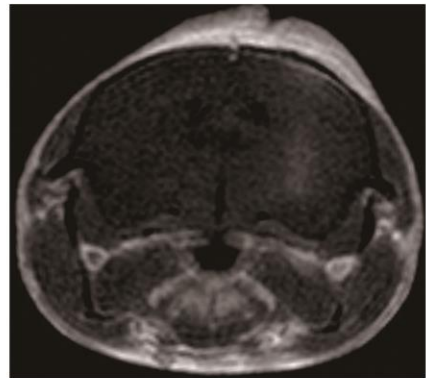

Mouse 3

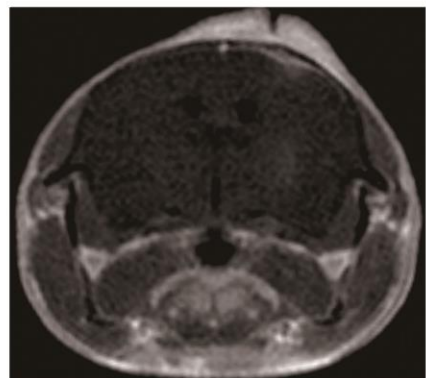

Supplement: Supplementary file 1 [file vaccines-11-01275-s001.zip › vaccines-2518291-supplementary.pdf]
